# Deep Sequencing of the Human Retinae Reveals the Expression of Odorant Receptors

**DOI:** 10.3389/fncel.2017.00003

**Published:** 2017-01-24

**Authors:** Nikolina Jovancevic, Kirsten A. Wunderlich, Claudia Haering, Caroline Flegel, Désirée Maßberg, Markus Weinrich, Lea Weber, Lars Tebbe, Anselm Kampik, Günter Gisselmann, Uwe Wolfrum, Hanns Hatt, Lian Gelis

**Affiliations:** ^1^Department of Cell Physiology, Ruhr-University BochumBochum, Germany; ^2^Department of Cell and Matrix Biology, Johannes Gutenberg University of MainzMainz, Germany; ^3^Department of Ophthalmology, Ludwig Maximilian University of MunichMunich, Germany

**Keywords:** odorant receptors, retina, next-generation sequencing, transcriptome, immunohistochemistry (IHC)

## Abstract

Several studies have demonstrated that the expression of odorant receptors (ORs) occurs in various tissues. These findings have served as a basis for functional studies that demonstrate the potential of ORs as drug targets for a clinical application. To the best of our knowledge, this report describes the first evaluation of the mRNA expression of ORs and the localization of OR proteins in the human retina that set a stage for subsequent functional analyses. RNA-Sequencing datasets of three individual neural retinae were generated using Next-generation sequencing and were compared to previously published but reanalyzed datasets of the peripheral and the macular human retina and to reference tissues. The protein localization of several ORs was investigated by immunohistochemistry. The transcriptome analyses detected an average of 14 OR transcripts in the neural retina, of which *OR6B3* is one of the most highly expressed ORs. Immunohistochemical stainings of retina sections localized OR2W3 to the photosensitive outer segment membranes of cones, whereas OR6B3 was found in various cell types. OR5P3 and OR10AD1 were detected at the base of the photoreceptor connecting cilium, and OR10AD1 was also localized to the nuclear envelope of all of the nuclei of the retina. The cell type-specific expression of the ORs in the retina suggests that there are unique biological functions for those receptors.

## Introduction

The odorant receptors (ORs) provide the basis for the detection of volatile odorant molecules in the environment. OR genes were previously determined to be expressed exclusively in the olfactory epithelium (Buck and Axel, 1991). However, recent studies have demonstrated that the expression of the OR genes occurs beyond the olfactory epithelium, such as in the heart, the prostate and the liver (Feldmesser et al., 2006; Zhang et al., 2007; Flegel et al., 2013). Nevertheless, the physiological function of the ORs in non-olfactory tissues remains widely unknown. The first report of a human OR functioning outside the olfactory epithelium revealed that the ORs are important for chemotaxis in sperm (Spehr et al., 2003). Furthermore, different studies have demonstrated the functionality of ectopically expressed ORs indicating that there is a diagnostic and a therapeutic potential of this receptor family in humans. For example, in the development of cancer diagnostics, OR51E2 has been described as a potential biomarker for the identification of prostate cancer due to its upregulation in prostate carcinoma cells compared to healthy prostate epithelial cells (Xu et al., 2000; Xia et al., 2001). Further studies have shown that the ORs have the potential to function as targets for therapeutic applications. In this context, the activation of OR51E2 by its specific agonist β-ionone leads to the inhibition of prostate carcinoma cell proliferation (Neuhaus et al., 2009). OR2AT4 is another example of the therapeutic potential of ORs in human skin because its activation promotes wound healing processes in human keratinocytes (Busse et al., 2014). They are also of major importance for the regulation of apoptosis, cytokinesis, hormone secretion and differentiation (Braun et al., 2007; Zhang et al., 2012; Maßberg et al., 2015; Gelis et al., 2016; Manteniotis et al., 2016a,b; Massberg et al., 2016; Tsai et al., 2016). However, the expression and localization of ORs in different areas of the brain, the spinal cord, the trigeminal (TG) and dorsal root ganglia (DRG) were recently described (Otaki et al., 2004; Garcia-Esparcia et al., 2013; Flegel et al., 2015; Goncalves et al., 2016). Yet, the physiological function of ORs in the nervous system remains elusive. The expression of ORs in parts of the eye has recently been proven, however, until now, no study has investigated the expression of ORs in neuronal cells of the retina. Pronin et al. (2014) were the first to characterize the expression of ORs and related genes in the mouse corneal transcriptome, whereas Ma et al. (2015) identified the expression of OR2W3 in retinal pigmented epithelium cells.

In the current study, we are the first to describe the complete expression profile of the ORs in the human retina by RNA-Sequencing (RNAseq) analysis using next-generation sequencing. We also validated the protein expression and the protein localization of several ORs by immunohistochemistry. The identification of the ORs expressed in the human retina provides the basis for the further functional characterization and potential application as therapeutic targets.

## Materials and Methods

### Ethics Statement

The RNAseq analysis of human retinae was conducted with anonymized probes from three different donors. For the western blotting analysis and the immunohistochemistry, human donor eyes (donor number 199, female, age 66 and 252, female, age 68) with no history of retinal disease were obtained from the Department of Ophthalmology, University Medical Center Mainz, Germany and Department of Ophthalmology, Ludwig Maximilian University, Munich, Germany. We followed the guidelines of the declaration of Helsinki and the study was approved by the local ethics boards of the clinical and the experimental study contributors (Nr. 331-09).

### Transcriptome Analysis

For the transcriptome analysis, the RNAs from the human retinae were isolated using the RNeasy Plus Mini Kit (Qiagen, Hilden, Germany) according to the manufacturer’s protocol, including the DNaseI digestion. The mRNA isolation from total RNA and sequencing analysis was performed by *GENterprise* Genomics (Mainz, Germany) using the Illumina NextSeq500 sequencing platform as paired-end reads with a 101-nucleotide (dataset Retina2) or with a 151-nucleotide (datasets Retina1 and Retina3) length (Supplementary Table S1). We analyzed the mRNAseq data as described previously (Flegel et al., 2013). For the detection of uniquely mapping reads with bowtie, the multi read parameter -m was set to 1. Prior to analysis, the read length was trimmed to 100 nt to ensure better comparability of the three datasets. The raw sequence data were aligned to the human reference genome hg19 using TopHat (Trapnell et al., 2009). Bowtie, the ultra-fast short-read mapping program, served to arrange the alignment (Langmead et al., 2009). The BAM-files were sorted and indexed using the Samtools software package (Li et al., 2009). The FPKM (fragments per kilobase of exon per million fragments mapped) values were calculated using Cuﬄinks (Trapnell et al., 2010). We reanalyzed previously published raw data in the same manner to compare with the data newly generated for this study. We used datasets from the macular and the peripheral retinae samples (Li et al., 2014) and from the human fetal retinal pigment epithelium (RPE) that were available in the NCBI SRA archive under the following accession numbers: peripheral retina (SRR1067928, SRR1067936, SRR1067942, SRR1067948 and SRR1067984), anatomical macular retina (SRR1067929, SRR1067939, SRR1067944, SRR1067970 and SRR1067986) and human fetal RPE (SRR447138). The peripheral and the macular retina datasets were summarized, and the expression data were presented as the means of the FPKM values (mFPKM). The differential expression analyses between the peripheral and the macular datasets were performed with Cuffdiff, the Cuﬄinks application (Trapnell et al., 2012). Six of the eight reference tissues were obtained from the Body Map 2.0 project from the NCBI GEO database^1^ (accession number: GSE30611), as previously described (Flegel et al., 2013). The human TG and the DRG raw data were taken from an earlier study by Flegel et al. (2015). The FPKM values of four TG datasets were combined and presented as the mFPKM. The human olfactory epithelium raw data were taken from (Olender et al., 2016). The data were bound and presented with the mean transcripts per million (TPM) of the four samples. We calculated the TPM values also for retina and reference tissues to enable a further meaningful possibility to compare the expression level between the different tissues (Wagner et al., 2012). All datasets were equivalently analyzed with the same parameters. The datasets were visualized and investigated by the Integrative Genomic Viewer (IGV)^2^ for proving sequence alignments and for the correct mapping of reads for the top expressed genes. While the raw data analysis was performed on a Linux based computer, further calculations were carried out with SigmaPlot 12.3 (Systat Software Inc., San Jose, CA, USA).

To determine a FPKM cutoff value for OR expression, we estimated the background of detection. For this purpose, we used modified gtf-files where the annotated transcripts of the ORs were shifted to random positions in the intergenic regions basically as described by Flegel et al. (2013). Mapping of fragments to these positions was considered as possible background. In the range of FPKM > 0.1, 21.7 OR genes were detected in average in the three retina sequencings and 1.3 in the random OR analysis, a fraction of 6% (Supplementary Figure S1). This percentage drops to 2.5% in the range of 0.1–0.3. However, considering all FPKM values >0.3, no ORs were detected using the gtf-files with the random positions. Therefore, we are confident that it is sensible to set the value for OR detection to 0.3 FPKM.

### Reverse Transcription Polymerase Chain Reaction

The total RNA from the retinae was reversely transcribed using the iScript cDNA Synthesis Kit (Bio-Rad Laboratories, Hercules, CA, USA) according to the manufacturer’s instructions. The equivalent of approximately 50 ng of RNA was used for each of the RT-PCR experiments. We designed primers that detect approximately 150–450 bp fragments of the OR open reading frame (ORF; Supplementary Table S2). The PCR was performed under standard PCR-conditions with the Mastercycler ep Gradient S (Eppendorf, Hamburg, Germany; 20 μl total volume, 40 cycles: 95°C, 59°C, 72°C, 45 s each). All experiments were conducted in triplicate.

### Antibodies

The polyclonal antibody rabbit anti-OR6B2/3 was purchased from Novus Biologicals (NBP1-71360, Cambridge, UK). It was generated against a synthesized C-terminal peptide derived from human OR6B2. The polyclonal rabbit antibodies against OR2W3, OR5P3, and OR10AD1 were obtained from Sigma-Aldrich (St. Louis, MO, USA; HPA045594, HPA060787, HPA049913). The specificity of OR antibodies was verified using recombinantly expressed rho-tagged ORs in Hana3A cells (Supplementary Figure S2; Flegel et al., 2016). As markers for cell compartments following antibodies were applied: monoclonal mouse antibodies against centrin-3 as a marker for the connecting cilium and basal body complex of photoreceptor cells (Trojan et al., 2008) and antibodies against β-catenin (Santa Cruz, Heidelberg, Germany), for the Cis-Golgi GM130 (BD Transduction Laboratories, Heidelberg, Germany), calnexin (Abcam, Cambridge, UK), LAMP1 (Lysosomal-Associated Membrane Protein 1; BD Transduction Laboratories, Heidelberg, Germany), as a markers for the cell adhesions complexes of the outer limiting membrane (OLM), the Cis-Golgi, the ER, and lysosomes, respectively.

### Immunohistochemistry

The two donor eyes were dissected and fixed in melting isopentane (at -168°C) and then cryosectioned as described in Overlack et al. (2011). Sections were double stained with the mouse monoclonal anti-centrin-3 antibody (Trojan et al., 2008) or other molecular marker antibodies in combination with the affinity purified primary rabbit antibodies against the ORs (1:50), followed by the appropriate secondary antibodies conjugated to the Alexa488 or the Alexa568 or the Alexa555 (Molecular Probes^®^, Life Technologies, Darmstadt, Germany), or CF^TM^-640 (Biotrend Chemikalien GmbH, Cologne, Germany), respectively. Some of the samples were counterstained with the fluorescein-labeled lectin peanut agglutinin (FITC-PNA, 1:400, Sigma Aldrich/Vector laboratories, Burlingame, CA, USA), which specifically labels the extracellular matrix sheath of the cone photoreceptor cells (Blanks and Johnson, 1984; Reiners et al., 2005; Wunderlich et al., 2015) and with 4′,6-diamidino-2-phenylindole (DAPI; Sigma Aldrich), before mounting with Mowiol 4.88 (Roth, Karlsruhe, Germany). Analysis was performed using a Leica DM 6000 B microscope (Leica microsystems, Bensheim, Germany). Images were processed, with respect to brightness, contrast, maximal projections of z-stacks, and picture size, including bicubic pixel interpolation, using the Leica deconvolution software and Adobe Photoshop CS (Adobe Systems, San Jose, CA, USA).

## Results

**FIGURE 1:**
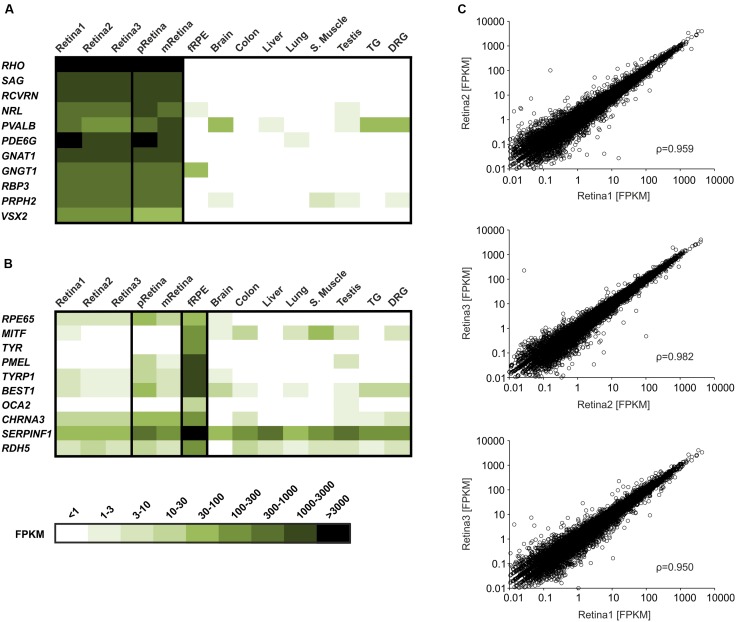
**Comparison of the RNA-Sequencing (RNAseq) data from the retina samples and from the reference tissues. (A)** Expression of the retinal marker genes: Rhodopsin (*RHO*), S-arrestin (*SAG*), recoverin (*RCVRN*), neural retina-specific leucine zipper protein (*NRL*), parvalbumin (*PVALB*), phosphodiesterase 6G (*PDE6G*), G protein subunit alpha transducin 1 (*GNAT1*), G protein subunit gamma transducin 1 (*GNGT1*), retinol-binding protein 3 (*RPB3*), peripherin 2 (*PRPH2*) and visual system homeobox 2 (*VSX2*). The heat map shows fragments per kilobase of exon per million fragments mapped (FPKM) values of the retina marker proteins in three whole retina samples compared to the peripheral retina (pRetina) and the macular retina (mRetina) samples and to the reference tissues [human fetal RPE (fRPE), brain, colon, liver, lung, skeletal (S.) muscle, testis, trigeminal (TG) and dorsal root ganglia (DRG)]. **(B)** Expression of the retinal pigment epithelium (RPE) marker genes: RPE-specific 65 kDa protein (*RPE65*), microphthalmia-associated transcription factor (*MITF*), tyrosinase (*TYR*), premelanosome protein (*PMEL*), tyrosinase-related protein 1 (*TYRP1*), bestrophin-1 (*BEST1*), oculocutaneous albinism II (*OCA2*), cholinergic receptor nicotinic alpha 3 subunit (*CHRNA3*), pigment epithelium-derived factor (*SERPINF1*) and 11-cis retinol dehydrogenase (*RDH5*). The heat map shows the expression of the RPE marker genes in three whole retina samples compared to the peripheral retina and the macular retina samples, the fRPE and the reference tissues (brain, colon, liver, lung, skeletal muscle, testis, TG and DRG). The darker colors indicate higher FPKM values and the white indicates the absence of any detectable transcripts. **(C)** The correlation of the FPKM values between the three human retina datasets. The Pearson product moment correlation values coefficients (ρ) are indicated and the *P* values are less than 0.001.

We used mRNAseq to characterize the transcriptomes of three individual human retinae from healthy donors. In this study, we sequenced up to nearly 34 million reads per sample. The data were analyzed with TopHat and Cuﬄinks software, and the reads were mapped to the human reference genome (hg19). The quantitative expression values were calculated for each sample based on the number of FPKM. In total, we detected the expression of approximately 17,100 genes (FPKM ≥ 0.1) of the 22,711 genes represented in the gene model. On a rough scale, a FPKM ≥ 0.1 corresponds to a weak expression level, a FPKM ≥ 10 represents a moderate expression level and a FPKM ≥ 100 indicates a high expression level. For example, the weakly to moderately expressed TATA box binding protein (*TBP*) is detected at 7–9 FPKM in the retina samples, whereas the strongly expressed glyceraldehyde-3-phosphate-dehydrogenase (*GAPDH*) gene reveals an expression value of approximately 2700 FPKM (Supplementary Figure S3). For analyses of the sample integrity, we calculated the FPKM values for the genes that were typically and specifically expressed in the neural retina (**Figure 1A**). To test whether the retina was dissected free of contamination with RPE, we evaluated the expression of typical RPE associated gene transcripts, which were relatively weakly or not at all expressed in Retina1–3 (**Figure 1B**). For example, the strongly expressed rhodopsin gene (*RHO*) had an expression value of ∼3,300–4,200 FPKM (**Figure 1A**). As shown in **Figure 1A**, the common neural retina marker genes are exclusively expressed in our generated retina samples. In comparison, the typical RPE-associated gene transcripts are relatively weakly or not expressed in Retina1–3 (**Figure 1B**). We further tested the variability of the human retinae transcriptome datasets using the Pearson product moment correlation and identified high correlations (ρ = 0.950–0.982) within our generated retina data (**Figure 1C**) and also between our data and the published data of the peripheral (pRetina; ρ = 0.848; Supplementary Figure S4A) and the macular retinae (mRetina; ρ = 0.793; Supplementary Figure S4B). Regarding the range of all determined FPKM values between 0 and 30 there is a high correlation between retina1–3. (ρ = 0.950–0.961; Supplementary Figure S5). The expression of ORs in human retina was in the same range. The expression pattern of all putative OR transcripts in retina1–3 showed relations as well (ρ = 0.972–0.994).

### Expression Analysis of the Odorant Receptors

**FIGURE 2:**
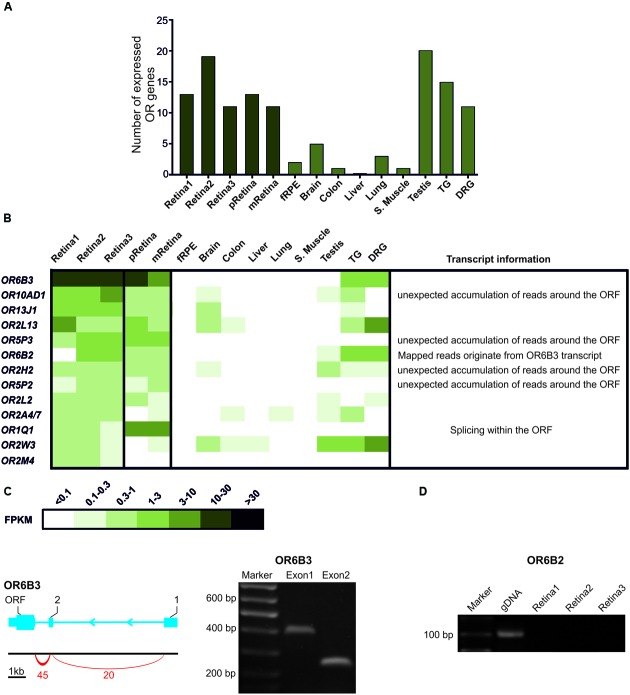
**The OR expression in the human retinae. (A)** The bar diagram shows the number of potential OR transcripts that are expressed with a FPKM value ≥0.3. For the pRetina, the mRetina and the TG, mFPKM values ≥0.3 were considered. **(B)** The heat map shows the FPKM values for the most abundant ORs (mean FPKM ≥0.3) compared to the peripheral retina and the macular retina samples and the reference tissues [fetal RPE (fRPE), brain, colon, liver, lung, skeletal (S.) muscle, testis, trigeminal (TG) and dorsal root ganglia (DRG)]. The darker colors indicate higher FPKM values and the white indicates the absence of any detectable transcripts. The ORs were sorted according to the mean FPKM found in the Retina1–3 (from healthy donors 1–3). **(C)** The transcript structure of the *OR6B3* was identified in human retinae. The left panel shows the schematic representations of the *OR6B3* transcript. The gene is indicated by the blue bars (exon) and the thin lines (intron). The coding exon is indicated by the ORF (open reading frame), the splice junctions with the red arcs and the arrows indicate the reading direction. The right panel shows the 5′UTR-validation of the OR-transcript using RT-PCR with intron-spanning primers in the Retina3 sample. The expression of the 5′UTRs of the OR transcript was confirmed by RT-PCR with a forward primer located in the identified exon and a reverse primer located in the ORF. *OR6B3*: Exon1 (forward primer in exon 1 of 5′UTR and reverse in *OR6B3* ORF); Exon2 (forward primer in exon 2 of 5’UTR and reverse primer in *OR6B3* ORF). The amplified PCR products were confirmed by Sanger sequencing. **(D)** Detection of *OR6B2* transcripts in retina 1–3 and genomic DNA (gDNA) as positive control by RT-PCR.

Next, we analyzed the expression of the OR transcripts in the human retinae compared to the reference tissue samples (**Figure 2**). Initially, we identified 11 to 19 different potential OR transcripts (for retina1 13, retina2 19 and retina3 11 transcripts) of 374 annotated OR genes in the three human retina datasets (FPKM ≥ 0.3; **Figure 2A**; Supplementary Table S3). Thereby, the number of expressed OR transcripts is in the same range as in the testis, the TG and the DRG (11–20 ORs), though higher than in the other investigated reference tissues (fRPE, brain, colon, liver, lung and skeletal muscle; 0–5 ORs). **Figure 2B** shows the OR expression profile sorted by the FPKM value of three whole retina samples from different donors in comparison to the available data from the pRetina and the mRetina (Li et al., 2014; the mFPKM values of five samples each). No significant difference in the pattern of OR transcripts between the pRetina and the mRetina from the same donor was observed (data not shown). The transcript properties and the additional transcript information of our retina datasets are shown in detail for the most highly expressed OR genes (mFPKM ≥ 0.3) in **Figure 2B**. In total, the expression of the eight potential OR transcripts was detectable across all analyzed retina samples, indicating a coherent donor-independent expression pattern of the highest transcripts. Furthermore, five OR transcripts were noticed in at least two different retina samples. Moreover, six of the eight donor-independent expressed OR transcripts were also identified in the mRetina and seven in the pRetina (mFPKM ≥ 0.3). The comparison of the OR expression profiles of the retina with the reference tissues revealed that the majority of potential OR transcripts are absent in reference tissues, excluding the TG and the DRG. Approximately 46% of the identified putative OR transcripts in the retina were also present in the analyzed TG and 30% in the DRG (mFPKM ≥ 0.3).

A detailed analysis of the transcript structures for the most highly expressed OR genes were performed by visualizing the read alignment of the RNAseq datasets using the IGV. *OR6B3* exhibited the highest expression of all of the investigated ORs (mFPKM ∼18). In addition to the retina, the *OR6B3* could also be detected in the TG and the DRG. Using the IGV, we confirmed the predicted expression of the *OR6B3* and also validated the expression of both of the 5′UTRs of *OR6B3* by RT-PCR with a forward primer located in the identified exon and a reverse primer located in the ORF of *OR6B3* (Flegel et al., 2015) (**Figure 2C**; Supplementary Figure S6). The ORFs of *OR6B3* and *OR6B2* are highly homologous (95% identical nucleotides). For *OR6B2*, reads are mapped only at the positions where the sequence is identical to *OR6B3* and virtually never at positions that were specific for *OR6B2*. Therefore, the sequenced reads originate from the *OR6B3* transcript and the expression of the *OR6B2* transcripts remains unclear. In addition, a detailed analysis of unique mapping reads indicates that *OR6B3* but not *OR6B2* is expressed in the human retina, which is also supported by RT-PCR analysis (**Figure 2D**, Supplementary Figure S7). For *OR2L13*, we detected the previously annotated 5′UTRs. For *OR2A7*, we observed the previously described exon sharing with *ARHGEF34P* [Rho guanine nucleotide exchange factor (GEF) 34, pseudogene; Flegel et al., 2013]. The tissue-dependent alternative usage of upstream exons has already been demonstrated (Asai et al., 1996; Flegel et al., 2013). The detection of annotated 5′UTRs and the corresponding exon-spanning reads is strong evidence for the presence of the OR-transcript. Furthermore, the visualization of *OR1Q1* revealed splicing events within their ORF, which are a typical feature of many of the OR-transcripts (Flegel et al., 2016).

Additionally, we also identified that the whole genome region around the ORF of *OR10AD1*, *OR2H2*, *OR5P2*, and *OR5P3* is evenly covered with reads and that no specific accumulation at the ORF is seen (Supplementary Figure S8). In these cases, the mapped reads and calculated FPKM values may partially originate from the overlapping transcripts of other non-annotated genes. Interestingly, according to the analyzed data, *OR5P3*, *OR5P2*, *OR1Q1*, and *OR2M4* are exclusively present in retina and not in any of the reference tissues. Using RT-PCR, we subsequently confirmed the presence of the putative OR transcripts in the human Retina1–3 that was shown in **Figure 2B** (Supplementary Figure S7). We further determined the TPM values for retina, olfactory epithelium and reference data for comparison of the expression levels between the tissues (Supplementary Table S4). This data verified the described expression pattern of ORs within the retina and relative to the reference tissues. Thus, *OR6B3* is also in term of TPM values the highest expressed OR in retina (TPM 43–57) and is in the reference tissues only detectable in TG and DRG.

### OR Protein Localization in the Human Retina

**FIGURE 3:**
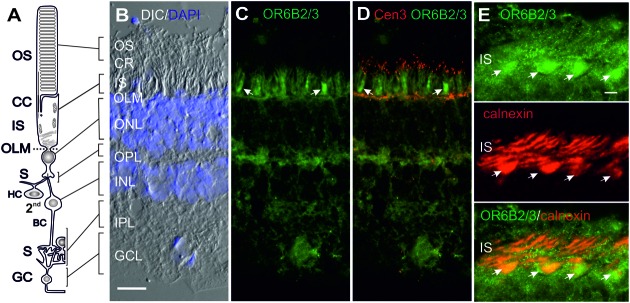
**Indirect immunofluorescence of OR6B2/3 in the human retina. (A,B)** A schematic diagram of a rod photoreceptor cell and the affiliated cells found in the retinal layers visualized in the 4′,6-diamidino-2-phenylindole (DAPI; blue) overlay of the differential interference contrast (DIC) image of a cryosection through the human retina. Indirect anti-OR6B2/3 (green) immunofluorescence in **(C)** and co-staining with the ciliary marker anti-centrin3 (Cen3, red) in **(D)** reveals OR6B2/3 localization in the inner segment (IS) of photoreceptor cells situated between the connecting cilium (CC) and the nuclei of the outer nuclear layer (ONL). Additional distinct anti-OR6B2/3 immunofluorescence is present in the outer plexiform layer (OPL) and in ganglion cells (GC) of the ganglion cell layer (GCL). Faint staining is present in the inner plexiform layer (IPL). **(E)** Double immunofluorescence of anti-OR6B2/3 and anti-calnexin, a marker for the endoplasmic reticulum (ER) demonstrated co-staining in the ER in the wider ISs of cone photoreceptor cells (arrows). Abbreviations: Photoreceptor outer segment (OS), outer limiting membrane (OLM), ciliary region (CR), secondary neurons (2nd), horizontal cells (HC), bipolar cells (BC) and synaptic regions (S). Scale bar represent 20 μm.

**FIGURE 4:**
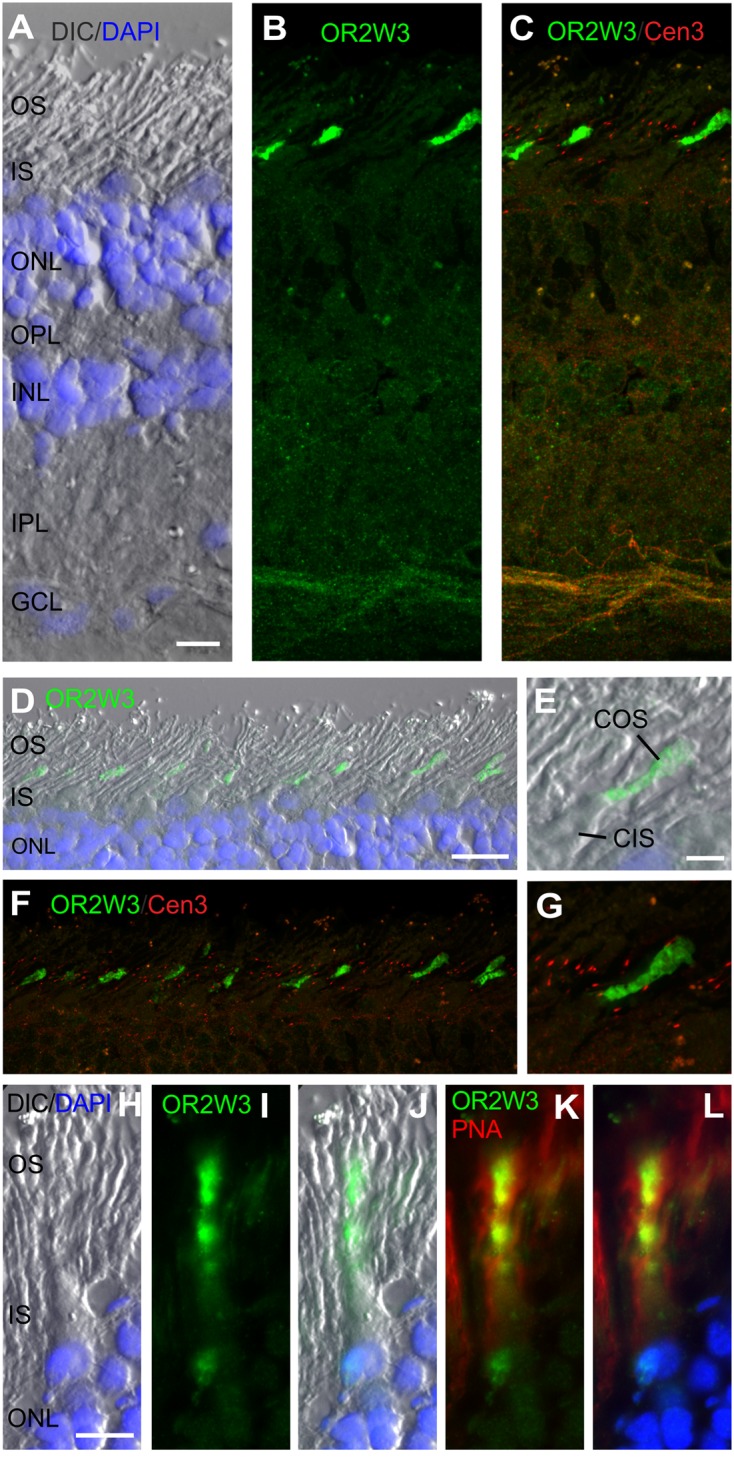
**Indirect immunofluorescence of OR2W3 in the human retina. (A–C)** Triple staining of a longitudinal section through a human retina. **(A)** A DIC image, merged with DAPI staining (blue), visualizes the retinal layers. The indirect immunofluorescence of anti-OR2W3 (green) in **(B)** is co-stained with the ciliary marker anti-Cen3 (red) in **(C)**, revealing the prominent localization of OR2W3 in a subset of photoreceptor cells. **(D–G)** Higher magnification images of the photoreceptor layer of a triple stained human retina indicates staining of the cone outer segments (COS) in the lower portion of the outer segment (OS) layer. **(H–L)** Counterstaining with the fluorescently labeled PNA (red), a molecular marker of the extracellular sheath of cones, affirmed that the OR2W3 (green) is present in the cone photoreceptor outer segments. Abbreviations: Inner segment (IS), outer nuclear layer (ONL), outer plexiform layer (OPL), inner nuclear layer (INL), inner plexiform layer (IPL), ganglion cell layer (GCL) and cone inner segment (CIS). Scale bar: 25 μm **(A–C)**, 20 μm **(D,F)**, 5 μm **(E,G)** and 10 μm **(H–L)**.

**FIGURE 5:**
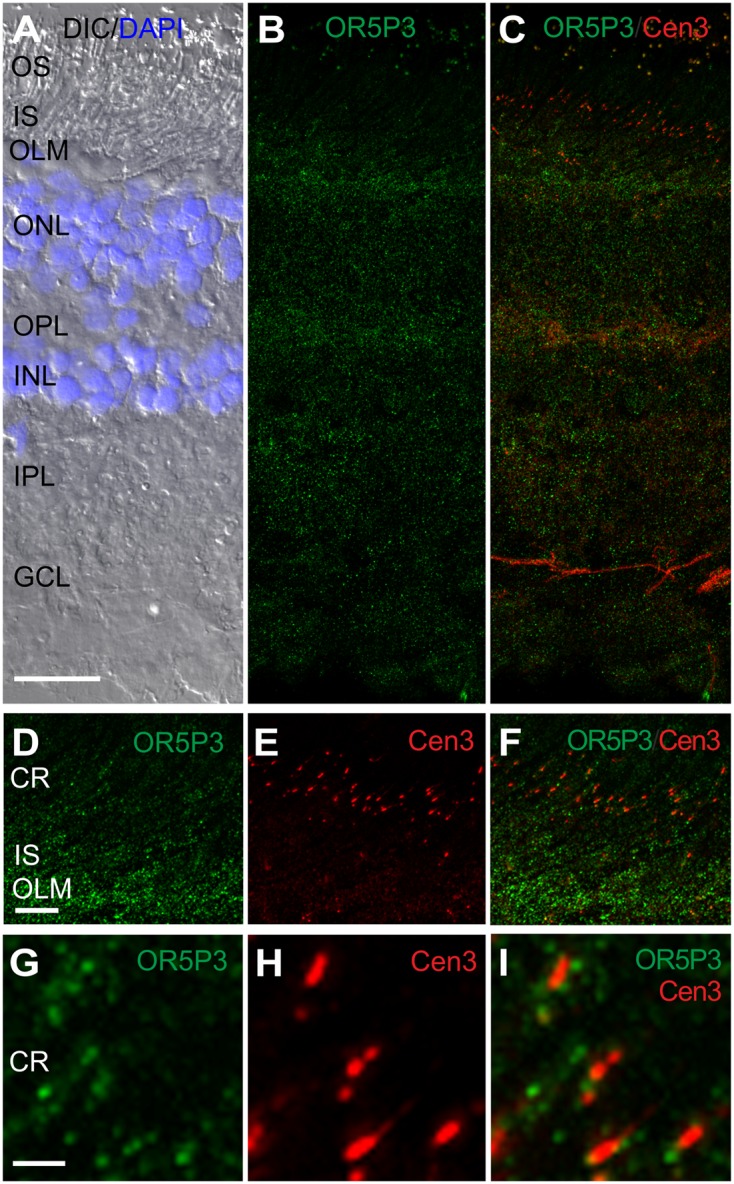
**OR5P3 localization in the human retina. (A–C)** Triple fluorescent staining shows anti-OR5P3, anti-Cen3 and DAPI in a longitudinal section of a human retina. **(A)** The DIC image, merged with DAPI staining (blue), visualizes the retinal layers. Anti-OR5P3 fluorescence (green) is present in almost all of the layers of the human retina, but more intensely in the OLM, the OPL and the INL. **(D–I)** Higher magnification images of the ciliary region (CR) of the human retina. Counterstaining with the ciliary marker protein centrin3 (Cen3, red) reveals an association between OR5P3 and the ciliary base. Abbreviations: Outer segment (OS), inner segment (IS), outer nuclear layer (ONL), inner nuclear layer (INL) and ganglion cell layer (GCL). Scale bar: 25 μm **(A–C)**, 5 μm **(E–G)**, 1 μm **(G–I)**.

**FIGURE 6:**
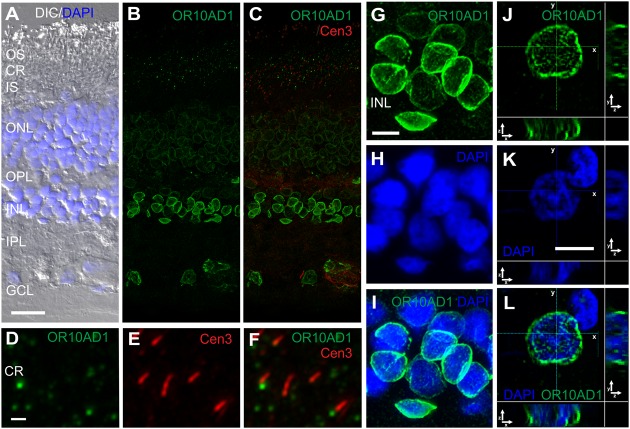
**OR10AD1 localization in the human retina. (A–C)** Triple fluorescent staining using anti-OR10AD1, anti-Cen3, and DAPI in a longitudinal section of a human retina. **(A)** The DIC image, merged with DAPI staining (blue), visualizes the retinal layers. **(B)** The indirect immunofluorescence of anti-OR10AD1 (green), merged with the indirect immunofluorescence of anti-Cen3 in **(C)**. **(D–F)** Higher magnification images of the CR of the photoreceptor layer illustrate that the OR10AD1 localizes at the base of photoreceptor cilia. **(G–I)** Higher magnification images of a part of the inner nuclear layer (INL). OR10AD1 is localized to the nuclear envelope of all of the nuclei of the human retina. **(J–L)** Z-scans of an anti-OR10AD1 stained nucleus demonstrating the localization of OR10AD1 in the nuclear envelope. In addition, OR10AD1-positive spots were found in the nucleus. Abbreviations: Outer segment (OS), inner segment (IS), outer nuclear layer (ONL) outer plexiform layer (OPL), inner plexiform layer (IPL) and ganglion cell layer (GCL). Scale bar: 20 μm **(A–C)**, 1 μm **(D–F)**, 5 μm (**G–I**,**J–L**).

We studied the subcellular localization of the selected ORs, according to availability of specific antibodies, *in situ* by using cryosections taken through the retinae of two mature donors (**Figures 3–6**). The well-defined layering of the human neural retina makes it relatively simple to determine the spatial distribution of proteins in a retinal section even by light microscopy (**Figures 3A,B**, **4A**, **5A** and **6A**). The indirect immunofluorescence staining of cryosections throughout the two human retinae from both donors was consistent and revealed the expression of all four ORs (OR6B2/3, OR2W3, OR5P3, and OR10AD1) in the human retina (**Figures 3C**, **4B**, **5B** and **6B**). To determine the subcellular localization, we counterstained the sections with the nuclear/DNA marker DAPI and with antibodies against diverse molecular markers of cell compartments (see section Materials and Methods). For example we used the antibody detecting centrin 3 as a well characterized marker for the connecting cilium, the basal body and the adjacent centriole of photoreceptor cell cilia at the junction of the inner and outer segment (OS; Trojan et al., 2008), thereby defining the photoreceptor compartments. Immunofluorescence staining using anti-OR6B2/3 revealed distinct labeling of the photoreceptor inner segments (ISs), of the outer plexiform layer (OPL) in which the photoreceptor cell synapses contact the secondary neurons of bipolar cells (BC) and horizontal cells (HC), and of ganglion cells (GC; **Figures 3C,D**). Faint staining was detected in the inner plexiform layer (IPL). Counterstaining with the antibody against calnexin, an ER marker, showed co-labeling in the photoreceptor IS, particularly in the wider cone IS (**Figure 3D**). Additionally, Western blotting analysis confirmed the presence of OR6B2/3 protein expression in the human retina (Supplementary Figure S9).

Indirect immunofluorescence staining using anti-OR2W3 resulted in intense immunofluorescence in a subset of photoreceptor cells (**Figures 4A–L**). Counter-staining with the anti-centrin 3 demonstrated the OS localization of OR2W3 in these photoreceptor cells (**Figures 4C,F,G**). The low number, the relative proximal position, and the morphology of the according cells (i.e., the rather thick IS) led us to the assumption that these cells were cone photoreceptors (**Figures 4A,B,D,E**). Counterstaining with a FITC-labeled peanut agglutinin (FITC-PNA), a classical marker for the cone photoreceptors specifically staining the extracellular sheath (Blanks and Johnson, 1984), affirmed the localization of OR2W3 in the cone outer segments (COS; **Figures 4H–L**).

We found punctate staining of the anti-OR5P3 in all of the layers of the human retina with lower concentrations at the OLM and the OPL (**Figure 5**). In the photoreceptor layer, OR5P3 was present in the ciliary region (CR) and in the IS of photoreceptor cells (**Figures 5D–F**). Higher magnification and co-staining with the ciliary marker anti-Cen3 demonstrated the association of OR5P3 with the ciliary base of photoreceptor cells (**Figures 5G–I**).

We detected OR10AD1 in the CR and in the nuclei of the outer and the inner nuclear layer, as well as in the GC of the human retina (**Figures 6A–C**). At higher magnification, co-staining with anti-centrin 3 demonstrated that OR10AD1 associates with the base of the photoreceptor cilia (**Figures 6D–F**). An overlay of the indirect immunofluorescence of anti-OR10AD1 with the fluorescence of the nuclear DNA marker DAPI revealed that the OR10AD1 localizes in the nuclear envelope of all of the nuclei in the human retina (**Figures 6G–I**). Interestingly, we observed that the most intense indirect immunofluorescence of anti-OR10AD1 associated with the nuclei of the IPL where the pericarya of the bipolar, horizontal and amacrine cells are located. An additional association with nuclei of photoreceptor cells in the outer nuclear layer was occasionally observed (data not shown). Z-scans of stained nuclei revealed that OR10AD1 is predominantly localized in the nuclear envelope and to a less extend in spots within the nucleus (**Figures 6J–L**).

## Discussion

Several studies have demonstrated the expression of ORs in various human non-olfactory tissues (Feldmesser et al., 2006; Zhang et al., 2007; Flegel et al., 2013). These findings have served as a basis for functional studies that demonstrate the potential of the ORs as drug targets (Neuhaus et al., 2009; Busse et al., 2014; Maßberg et al., 2015; Manteniotis et al., 2016a,b; Tsai et al., 2016). In this study, for the first time, we describe the expression of the OR mRNAs and the localization of the OR proteins in the mature human retina. We generated three RNAseq datasets of three individual human retinae, analyzed the transcriptomes and compared our data to the reanalyzed RNAseq datasets of the peripheral and the macular human retina from the NCBI SRA archive (Li et al., 2014), as well as to reference tissues. The data revealed that an average of 14 putative OR transcripts with an mFPKM value ≥0.3 are expressed in the human retina. Eight of the ORs (OR6B3, OR10AD1, OR13J1, OR2L13, OR5P3, OR2H2, OR2L2, and OR2A4/7) were constantly expressed in all of the three for this study generated retina datasets. Our results indicate a conserved OR expression pattern in the human retina as has been described for the olfactory epithelium, the TR/DRG and the spermatozoa/testis of humans (Fuessel et al., 2006; Flegel et al., 2015, 2016). The expression profile of ORs in retina and TG/DRG are very similar. Several putative OR transcripts were also detectable in brain and testis, whereby recently published RNAseq data revealed that only five ORs (OR2A4/7, OR2L2, OR2M4 OR2W3 and OR5P3; mFPKM value ≥0.3) were detectable in human olfactory epithelium (Olender et al., 2016). This finding implies that the function of ORs expressed in the retina but not found in olfactory epithelium cannot be directly connected to the sense of smell. Interestingly, *OR13J1, OR2L13*, and *OR6B3* appear to be neuron-specific based on the fact that they were found predominantly in neuronal tissues, namely the neuronal retina as we show in the present study, the TG/DRG and the brain (Flegel et al., 2015). In this context, OR2L13 is not only expressed in brain, it is also dysregulated in Parkinson’s disease (Garcia-Esparcia et al., 2013). The physiological function of ORs in the nervous system is far from understood, but according to the hypothesis by Otaki and Goncalves, ORs might play a role either in the detection of alterations in the chemical composition of cerebrospinal fluid, and therefore, the regulation of brain homeostasis or in developmental processes such as axon guidance and target recognition (Otaki et al., 2004; Goncalves et al., 2016).

Because the expression profile of ORs in the retina was not investigated until now, it was previously assumed that *OR6B3* were exclusively present in the TG and the DRG (Flegel et al., 2015). According to our current findings, we can extend the *OR6B3* expression profile to another sensory system, the retina. The assumption that this particular receptor has a vital function apart from olfaction is supported by the finding that its expression in the human olfactory epithelium is not clear (mFPKM 0.05; TPM 0.13; Olender et al., 2016).

The FPKM value of *OR6B3* in retina is between 16 and 21, whereas the FPKM values of the ORs expressed in the reference tissues are <6 and in olfactory epithelium <9.

In the case of the TG and the retina, *OR6B3* is also the most highly expressed OR in terms of its FPKM and TPM. In human, *OR6B3* belongs to the highest expressed ORs in terms of its FPKM and TPM values. The immunohistochemical staining of the human DRG indicated that OR6B3 (or OR6B2, respectively) is predominantly localized in the satellite glia cells (Flegel et al., 2015). In the human retina, we did not detect OR6B3 protein in glial cells, but in retinal neurons. It was detected in the photoreceptor IS, in the bipolar and horizontal cells and at the synapse between photoreceptor cells and the later second neurons in the OPL, as well as in the GC. Moreover, counterstaining revealed that OR6B3 is localized within the endoplasmatic reticulum. We suggest that the ER localization of OR6B3 is only a part of the trafficking process, whereby OR6B3 is translocated from cytosol to cytoplasm as required. It must be noted that the olfactory receptor-transporting proteins (*RTP1* and *RTP2*) were not detectable in human retina, only *REEP1*, which enhances the cell surface expression of ORs was expressed in retina according RNAseq data (Supplementary Figure S10). Flegel et al. (2013) analyzed the expression of canonical olfactory signaling pathway components in 16 different tissues and observed that REEP1 was expressed in various human tissues, whereas RTP1 or RTP2 were detected only in testis, breast, and skeletal muscle. Because the endogenous agonists for this receptor are unknown, no reliable conclusions concerning the functional role of OR6B3 can be drawn at this point in time. Nevertheless, the atypically high expression of an OR at the RNA and the protein level as well as the broad localization in the retina suggests a significant and general relevance.

A comparison between the pRetina and the mRetina showed that there were no significant differences in the OR expression pattern, implicating that the ORs are ubiquitously expressed in the human retina. However, OR2W3 is exclusively localized in the cone photoreceptors and had a higher expression level in the mRetina than in the pRetina. This is consistent with the high concentration of cones in the mRetina. OR2W3 is a broadly expressed OR and in some cases it was only found as a chimeric transcript with the E3 ubiquitin-protein ligase *TRIM58*, of unknown function (Flegel et al., 2013, 2016). However, in the data newly generated for this study, we observe only chimeric transcripts with *TRIM58* in one data set. The present immunohistochemical analyses revealed that the OR2W3 protein localizes in the OS of the cone photoreceptor cells. Similar to the olfactory cilia of the olfactory neurons, the photoreceptor OSs resemble specifically tuned primary sensory cilia (Fliegauf et al., 2007) harboring all of the components of the signal transduction cascade for their specific modality (Pugh and Lamb, 2000; DeMaria and Ngai, 2010). Therefore, as seven transmembrane domain proteins, OR2W3 and the cone opsins are most likely to be localized next to each other in the photosensitive OS membranes of the cones. It will be of particular interest to reveal if these GPCRs, cone opsins and OR2W3, may interact in their functions in the cone photoreceptors.

Furthermore, four OR genes (*OR5P3*, *OR5P2*, *OR1Q1*, and *OR2M4*) were found to be ectopically expressed exclusively in the human retina, suggesting a specialized function. One of them, OR5P3 is localized to the base of the photoreceptor connecting cilium, which corresponds to the transition zone of prototypic cilia (Roepman and Wolfrum, 2007) and controls ciliary import and export. In the periciliary region at the ciliary base, the transport complexes of the intraflagellar transport are assembled for cargo delivery into the cilium (Sedmak and Wolfrum, 2010; Lechtreck, 2015), which is essential for photoreceptor maintenance. OR5P3 may participate in this machinery within the ciliary base. Remarkably, almost one-quarter of the known photoreceptor degeneration genes are associated with ciliary structure or function (Kishida et al., 2007). OR5P3 has been previously de-orphanized (Saito et al., 2009; Li and Matsunami, 2011). Its activating ligands (+)-carvone and coumarin are natural substances that are contained in plants and are present in food, pharmaceuticals and cosmetics (Williams, 1973; Decarvalho and Dafonseca, 2006). Therefore, the de-orphaned OR5P3 could be an interesting target for functional studies. Furthermore, in certain cases, we observed that there was an unexpected accumulation of multiple reads around the ORF that has not been described up to date. We propose two alternative possibilities: (1) the OR gene is located in a cluster of unknown or unannotated genes in which transcripts overlap with the OR gene or (2) the OR gene is located downstream from a highly expressed gene in which termination was incomplete. We assume that the first option applies to *OR10AD1* and the second applies to *OR5P3*, *OR5P2*, and *OR2H2*, which does not exclude the possibility that the transcripts are functional. Moreover, we could detect OR5P3 and OR10AD1 at the protein level.

Immunohistochemical staining revealed that the orphan receptor OR10AD1 is associated with the photoreceptor cilia analog of OR5P3 and localized to the nuclear envelope of all of the nuclei of the human retina, whereby OR10AD1 was predominantly localized in the nuclear envelope and to a less extend in speckles within the nucleus of the inner nuclear layer. OR10AD1 was found in various tissues at the mRNA level, but its protein localization has not been studied (Flegel et al., 2013). Therefore, we could not conclude whether OR10AD1 is generally present at the nuclear envelope in many tissues or only in the retina.

The localization of GPCRs to the nuclear membrane has been shown for different cells and tissues. A few examples of class A subfamily of GPCRs, which also include the ORs, are the muscarinic acetylcholine receptor in the cornea, the α- and β-adrenergic receptors in cardiomyocytes and the apelin receptor in the cerebellum (Cavanagh and Colley, 1989; Buu et al., 1993; Lind and Cavanagh, 1995; Lee et al., 2004; Boivin et al., 2006; Huang et al., 2007; Vaniotis et al., 2011; Tadevosyan et al., 2012). This study first uncovered the presence of an OR in the nuclear envelope. To date, the presence of an OR beyond the cytoplasmic membrane was only described for OR2A4, which is localized to the cytokinetic structures of HeLa cells, and for other ORs in the sperm, melanocytes, and brain (Zhang et al., 2012; Flegel et al., 2016; Gelis et al., 2016; Garcia-Esparcia et al., 2013).

Most of the ‘classical’ downstream molecules that are associated with GPCR signaling at the plasma membrane, such as the G protein, adenylyl cyclase and Ca^2+^ channels, have also been found in the nucleus (Yamamoto et al., 1998; Zhang et al., 2001; Gobeil et al., 2002; Boivin et al., 2005; Bootman et al., 2009). Interestingly, the alpha-subunit of the G protein (Gα_olf_) canonically coupled to the ORs is also expressed in the photoreceptor cells (Zigman et al., 1993). Beside Gα_olf_, our RNAseq results revealed that from the members of the canonical olfactory downstream pathway also adenylyl cyclase III, two of three olfactory CNG channel subunits and the calcium-activated chloride channel were expressed in the human retina (Supplementary Figure S10). The detailed analysis of expression and localization of the downstream molecules is of interest for subsequent functional analyses. Worth mentioning is that Gα_olf_ is not only involved in the canonical olfactory receptor signaling but also mediates dopamine D1 receptor signaling in the striatum of the cortex (Zhuang et al., 2000). Thus, OR mediated signaling pathways may interact with the dopaminergic system in the retina, which regulates the circadian rhythms in the eye as a counterpart to the melatonin system. Dopamine receptors are also involved in cell survival and growth processes in the eye (Witkovsky, 2004) such as ectopically expressed ORs in prostate and keratinocytes (Massberg et al., 2016; Tsai et al., 2016). Interestingly, the expression level of dopamine receptors already described in retina such as D1 receptor (*DRD1*: mFPKM 9) is comparable to that of ORs (Supplementary Figure S11). Moreover, further GPCRs such as GABA and glutamate receptors, which participate in visual processing, or medium-wave-sensitive and short-wave-sensitive opsines are expressed in the same range as ORs (Supplementary Figure S11). Based on this observation, we assume that the OR expression in the human retina is sufficiently high to be an indicator of functionality. The fact that ORs are also weakly to moderate expressed in term of FPKM value in the olfactory epithelium, where they perform their main task in mediating olfaction, and in other cell types such as keratinocytes, where they are involved in growth processes, support this assumption (Olender et al., 2016; Tsai et al., 2016).

## Conclusion

For the first time, we have described the expression of ORs in the human retina. We identified OR6B3 as one of the highest ectopically expressed ORs in terms of its FPKM/TPM value and by its detection at the protein level. We observed that some ORs are expressed in diverse and other in specific cell types or regions of the retina. The specific localizations within the neural retina in general and on the subcellular level of the photoreceptor cells implicate the distinct functions of ORs outside of the olfactory epithelium (i.e., similar to the spermatozoa Flegel et al., 2016). For subsequent studies, an understanding of the functional role of the ORs in the retina would be of particular interest for the basic science community interested in cell signaling and sensory systems and may additionally offer opportunities for the development of novel therapies in the field of ophthalmology.

## Author Contributions

Wrote the paper: NJ, KW, LG, UW, and CH. Analyzed the data: NJ, KW, and GG. Designed the experiments: NJ, KW, GG, HH, LG, UW, and AK. Conducted the experiments: NJ, KW, LT, MW, CF, DM, and LW.

## Conflict of Interest Statement

The authors declare that the research was conducted in the absence of any commercial or financial relationships that could be construed as a potential conflict of interest.

## Footnotes

1 http://www.ncbi.nlm.nih.gov/gds/

2 http://www.broadinstitute.org/igv/
