# Supplementary material for: Deep Sequencing of the Human Retinae Reveals the Expression of Odorant Receptors
Source: Front Cell Neurosci. 2017 Jan 24;11:03. doi: 10.3389/fncel.2017.00003 (PMC5258773; doi:10.3389/fncel.2017.00003)
Supplement: Supplementary file 3 [file Data_Sheet_1.DOCX]

Supplementary Material

Deep sequencing of the human retinae reveals the expression of odorant receptors

Nikolina Jovancevic^1^*, Kirsten A. Wunderlich^2^, Claudia Haering^1^, Caroline Flegel^1^, Désirée Maßberg^1^, Markus Weinrich^1^, Lea Weber^1^, Lars Tebbe^2^, Anselm Kampik^3^, Günter Gisselmann^1^, Uwe Wolfrum^2^, Hanns Hatt^1#^ and Lian Gelis^1#^

^1^ Department of Cell Physiology, Ruhr-University Bochum, 44801 Bochum, Germany

^2^ Department of Cell and Matrix Biology, Johannes Gutenberg-University, 55099 Mainz, Germany

^3^ Department of Ophthalmology, Ludwig-Maximilians-University, 80336 Munich, Germany

*** Correspondence:**Nikolina Jovancevic, Department of Cell Physiology, Ruhr-University Bochum, 44801 Bochum, Germany; Nikolina.Jovancevic@rub.de

# Supplementary Figures and Tables

## Supplementary Figures


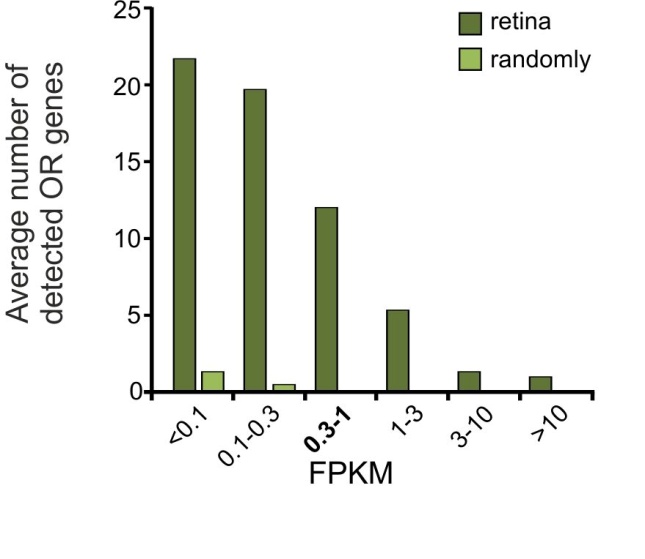


**Supplementary Figure S1.** Estimation of an expression threshold. Reads were mapped to exons (dark green color) and random positions in the intergenic regions (light green color). The expression levels of all genes and background regions of all three retina datasets were taken together. The genes expressed at levels under FPKM 0.1 are false positive in 6 % of cases and between 0.01 and 0.3 FPKM in 3 % of cases, whereas genes expressed at levels above 0.3 are 100 % true positive.

**
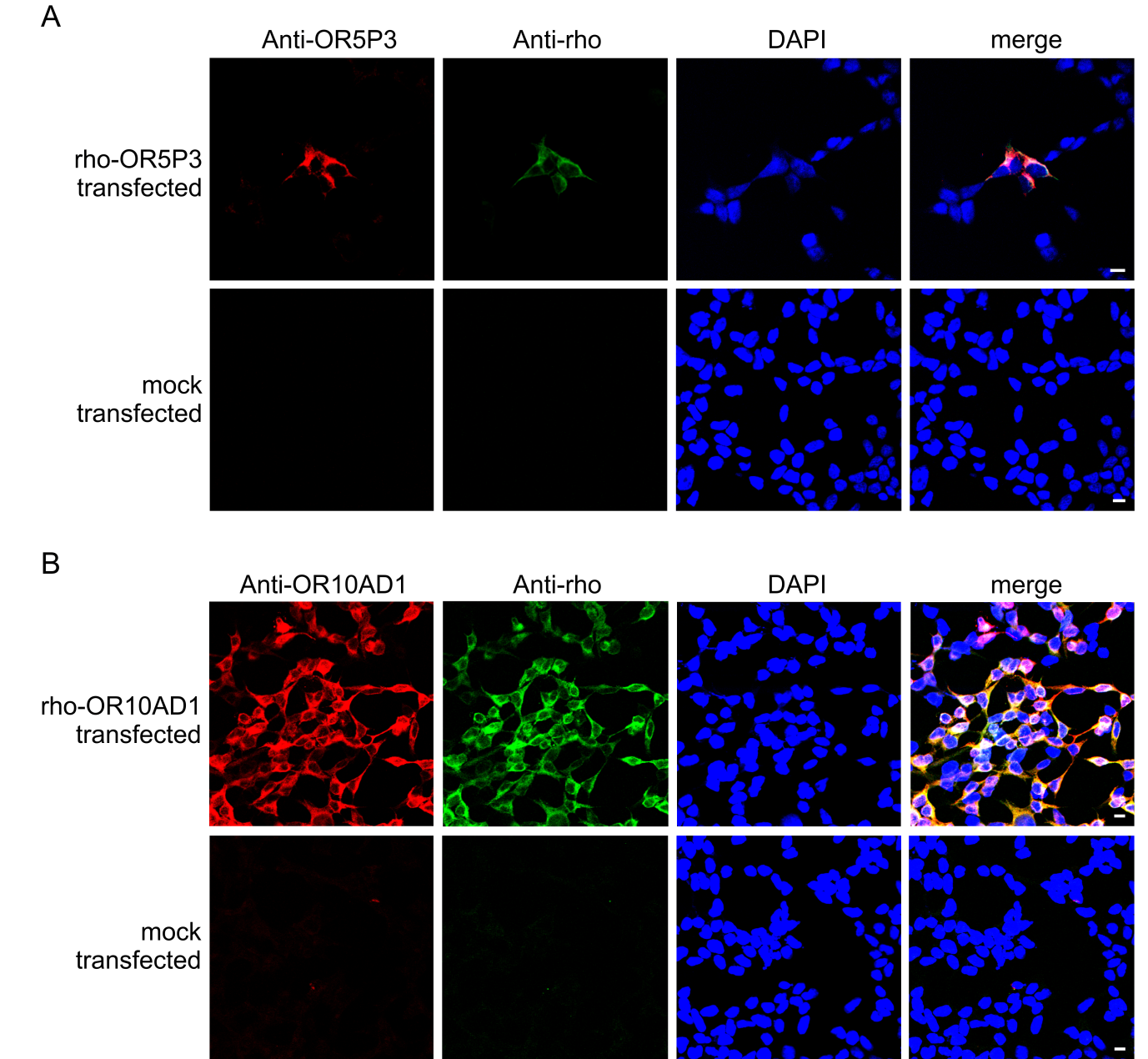
**

**Supplementary Figure S2.** The anti-OR antibodies specifically detect recombinantly expressed ORs in Hana3A cells. Co-immunostaining of Hana3A cells transiently transfected with the respective rho-tagged OR plasmid. Detection of the recombinant rho-OR5P3 **(A)** or rho-OR10AD1-protein **(B)** was performed using a specific antibody against the respective OR (green) and an antibody against the N-terminal rho-tag (red). Cell nuclei were stained with 4’,6-diamidino-2-phenylindole (DAPI). Mock-transfected Hana3A cells served as negative control. Bars indicate 10 µm.


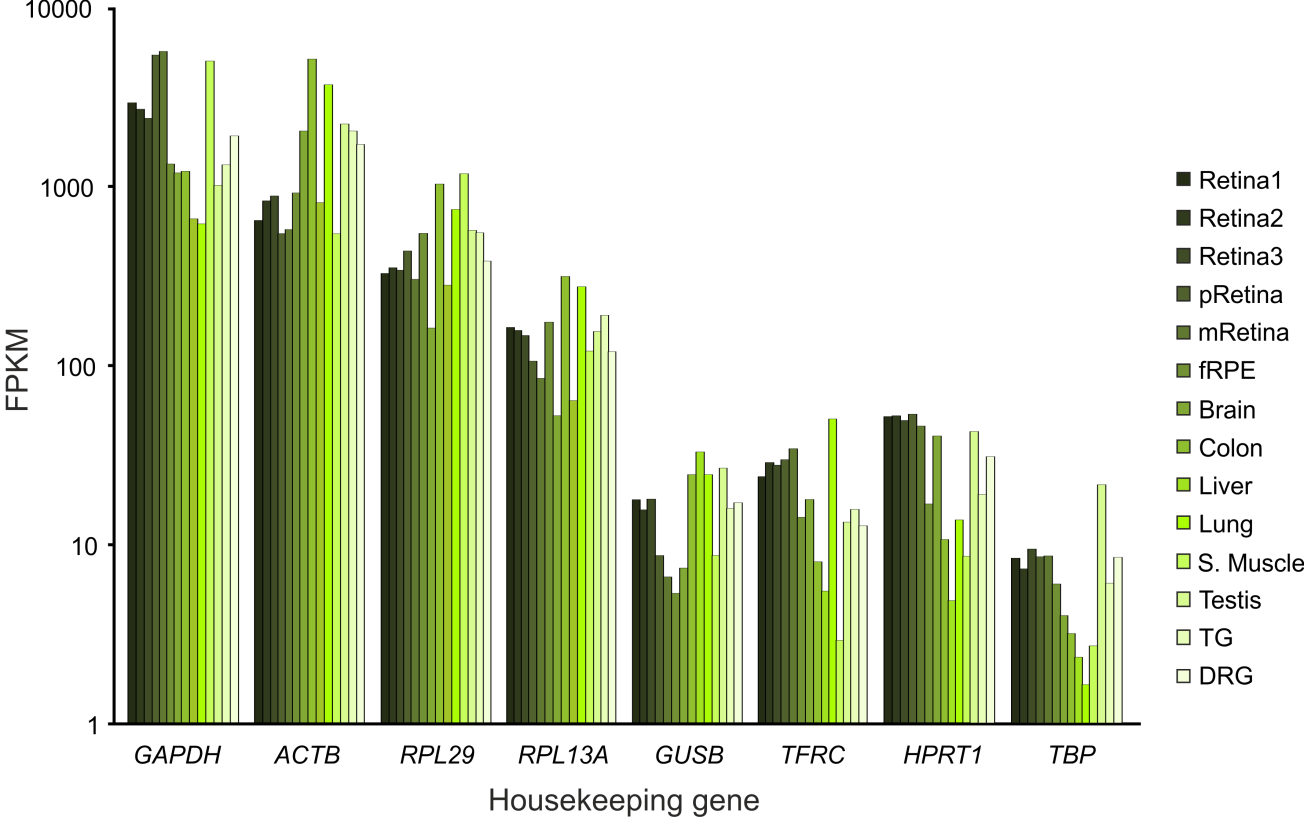


**Supplementary Figure S3.** The expression patterns of housekeeping genes in different human tissues. The housekeeping gene expression levels of the three retina samples, the peripheral retina (pRetina) and the macular retina (mRetina) and the reference tissues [human fetal RPE (fRPE), brain, colon, liver, lung, skeletal muscle (S. Muscle), testis, trigeminal (TG) and dorsal root ganglia (DRG)] are shown. The levels of the highly expressed genes glyceraldehyde 3-phosphate dehydrogenase (*GAPDH*) and β-actin (*ACTB*), the moderately to highly expressed genes ribosomal protein L29 (*RPL29*) and ribosomal protein L13A (*RPL13A*) and the weakly to moderately expressed genes β-glucuronidase (*GUSB*), transferrin receptor (*TFRC*), hypoxanthine phosphoribosyltransferase 1 (*HPRT1*) and TATA box binding protein (*TBP*) are included.


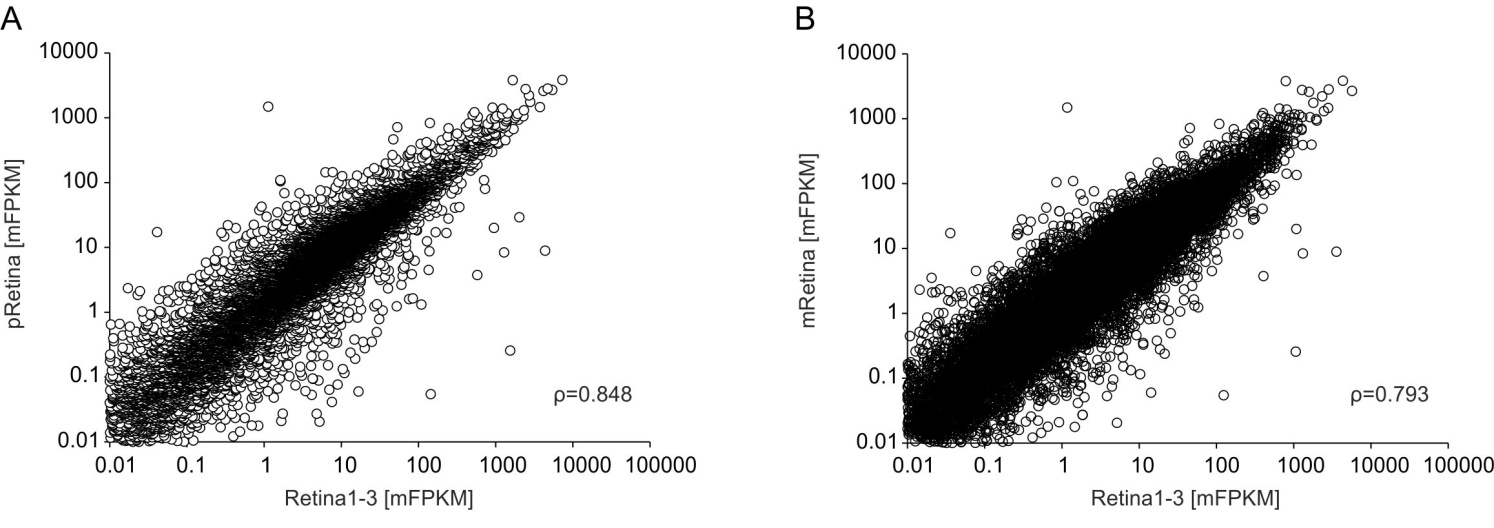
**Supplementary Figure S4.** Correlation of the mFPKM values of the three human retina datasets and peripheral retina (pRetina; **A**) or macular retina (mRetina; **B**). The Pearson product moment correlation coefficient (ρ) was calculated. P values are below 0.001.


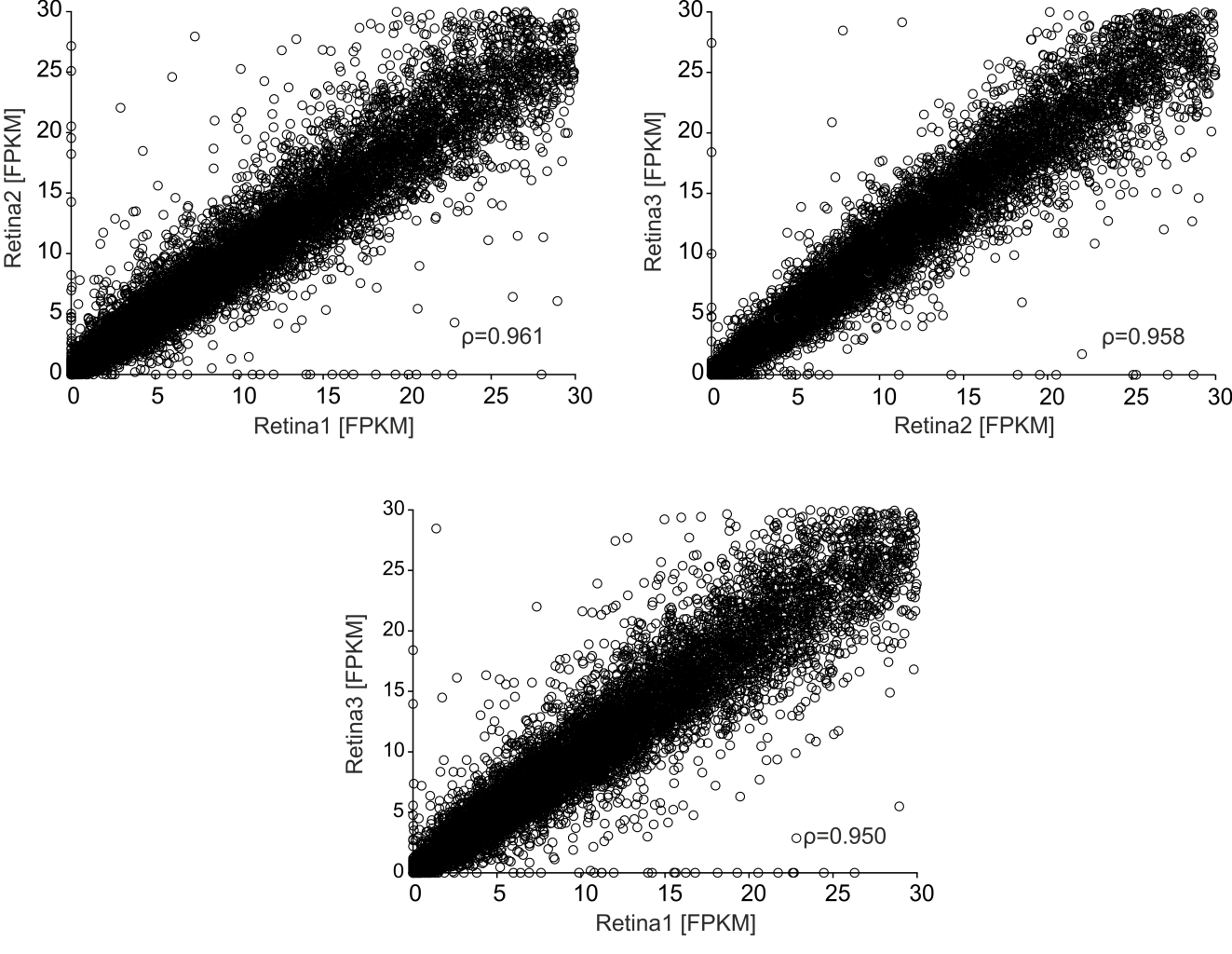


**Supplementary Figure S5.** The correlation of the FPKM values in the range between 0 and 30 of the three human retina datasets. The Pearson product moment correlation coefficient (ρ) was calculated. P values are below 0.001.


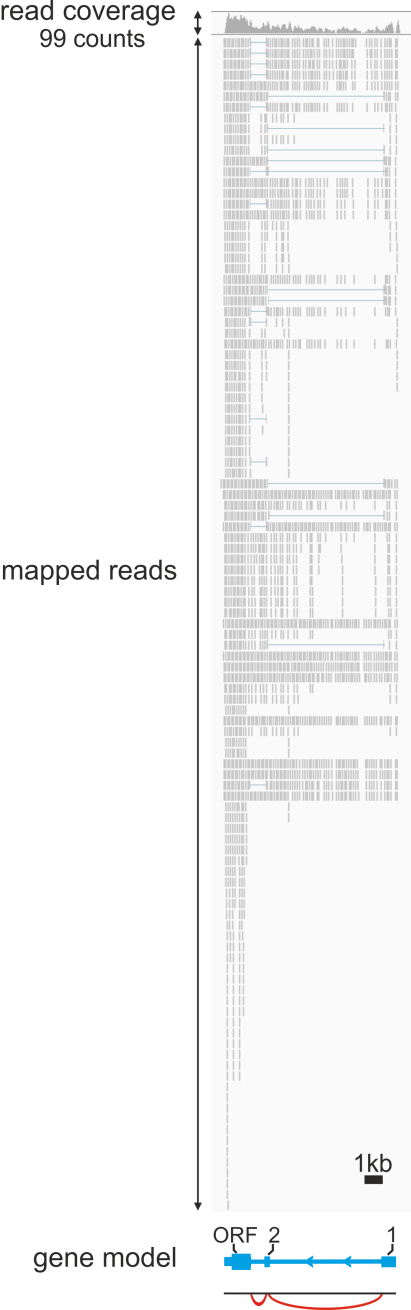


**Supplementary Figure S6.** The representation of read coverage of *OR6B3* in retina 3 (FPKM 21.08; Integrative Genomic Viewer). The gray segments indicate reads that were mapped onto the reference genome. The read coverage is shown above (detected and mapped counts/bases at each respective position). The lower panel shows the gene model of *OR6B3*. The gene is indicated by the blue bars (exon) and the thin lines (intron). The coding exon is indicated by the ORF (open reading frame), the splice junctions with the red arcs, and the arrows indicate the reading direction.


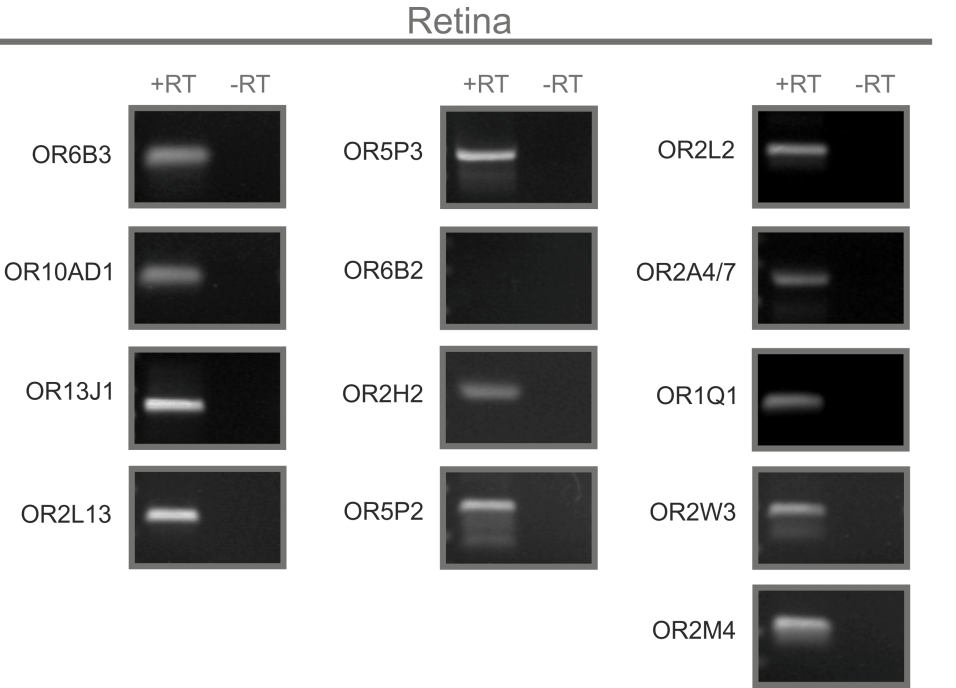


**Supplementary Figure S7.** RT-PCR validation of the RNAseq results on expression of the ORs. Gel electrophoresis of amplicons from the Retina2 cDNA (+RT) and the no reverse transcriptase controls (-RT) that exclude the possibility of genomic DNA contamination. The PCR results were verified by Sanger sequencing. In some instances, the primers amplified fragments that could originate from two highly homologous ORs. If that occurred, then both names were written.


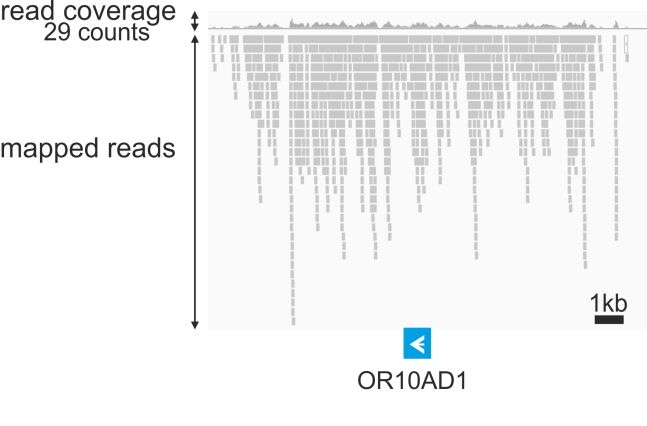


**Supplementary Figure S8.** *OR10AD1* is located within a cluster of reads. The representation of read coverage of the *OR10AD1* exon that was located in a highly expressed unidentified gene (Integrative Genomic Viewer). The gray segments indicate reads that were mapped onto the reference genome. The transcript is indicated by the blue bar (exon). The read coverage is shown above (detected and mapped counts/bases at each respective position).


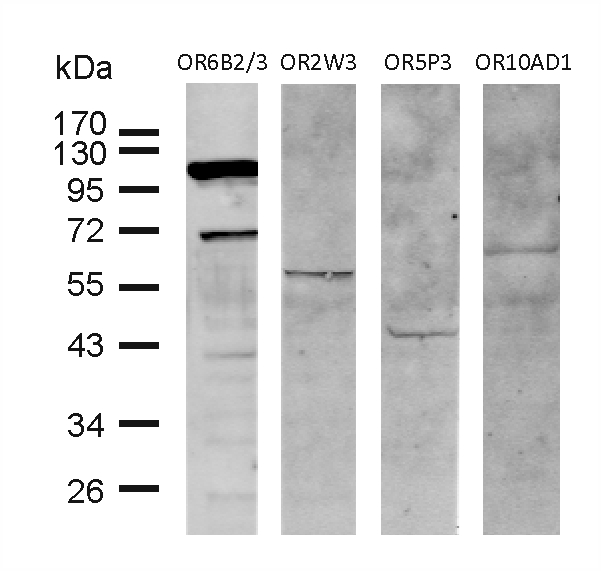


**Supplementary Figure S9.** Western blotting analysis reveals that OR6B2/3 protein is expressed in the human retina. Western blotting analysis of the protein extracts from the human retinal tissue using an antibody against the OR6B2/3 revealed that the OR6B2/3 protein is expressed. The predicted molecular weight of the OR6B3 monomeric protein is 37 kDa, the dimeric protein is 74 kDa and the trimeric protein is 111 kDa.

**
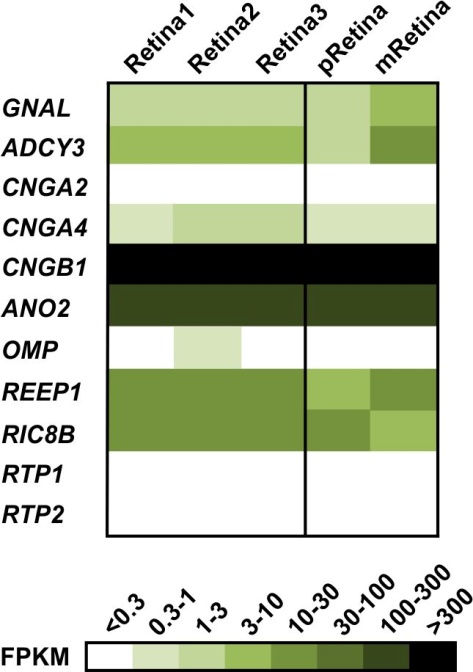
**

**Supplementary Figure S10.** Expression of canonical olfactory signaling pathway components in human retinae. Expression analysis of signaling components including Gα_olf_ (*GNAL*), adenylyl cyclase III (*ADCY3*), CNG channel subunits (*CNGA2*, *CNGA4* and *CNGB1*) and calcium-activated chloride channel (*ANO2*) and the nucleotide exchange factor Ric8b (*RIC8B*). We also analyzed the expression of the receptor-transporting proteins (*RTP1* and *RTP2*) and receptor-enhancing protein (*REEP1*) as well as the olfactory marker protein (*OMP*).

**
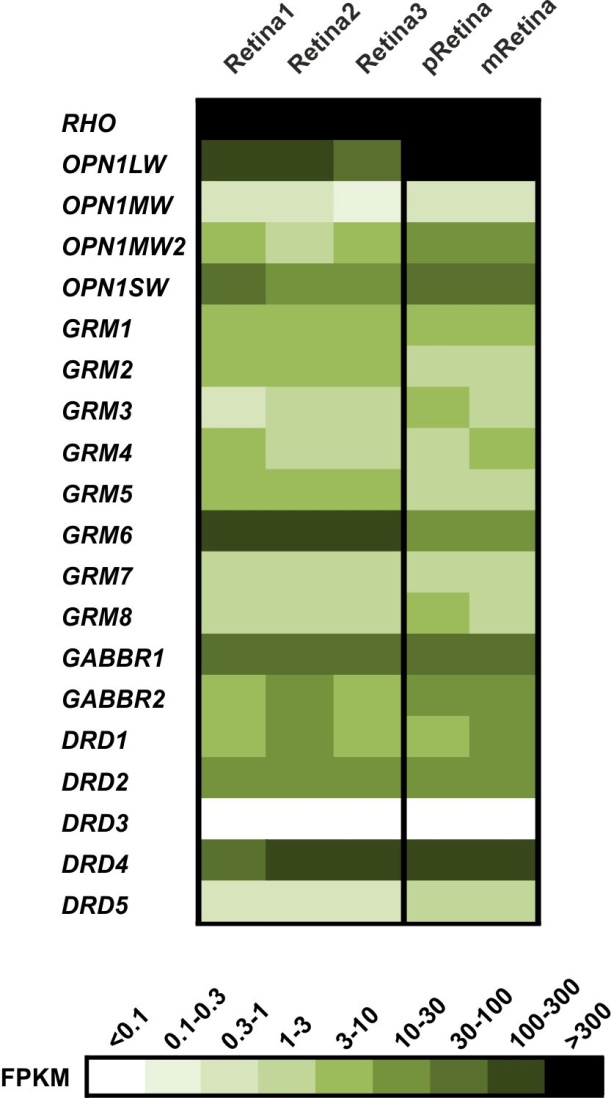
**

**Supplementary Figure S11.** Expression of GPCR in human retinae. Expression analysis of exemplarily chosen visual GPCR families including Rhodopsin (*RHO*), iodopsins (*OPN1LW*, *OPN1MW*, *OPN1MW2* and *OPN1SW*), glutamate receptors (*GRM1-5),* GABA receptors (*GABBR1* and *GABBR2*) and dopamine receptors (*DRD1-5)*

## Supplementary Tables

**Supplementary Table S1**. Sequencing details of RNAseq datasets of human retinae.


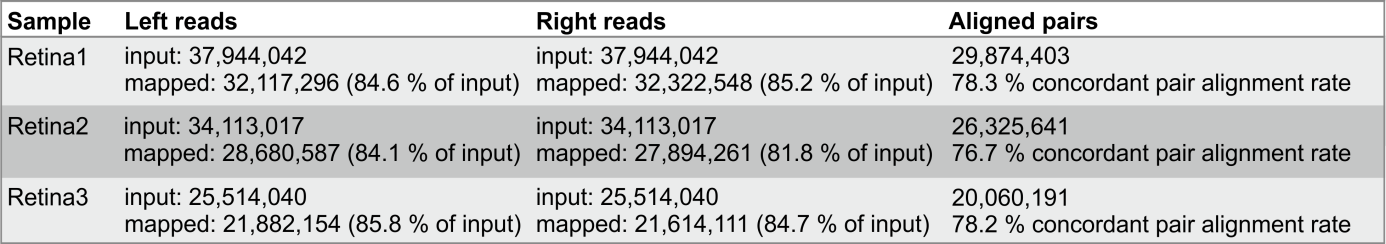


**Supplementary Table S2.** Primer sequences used for PCR


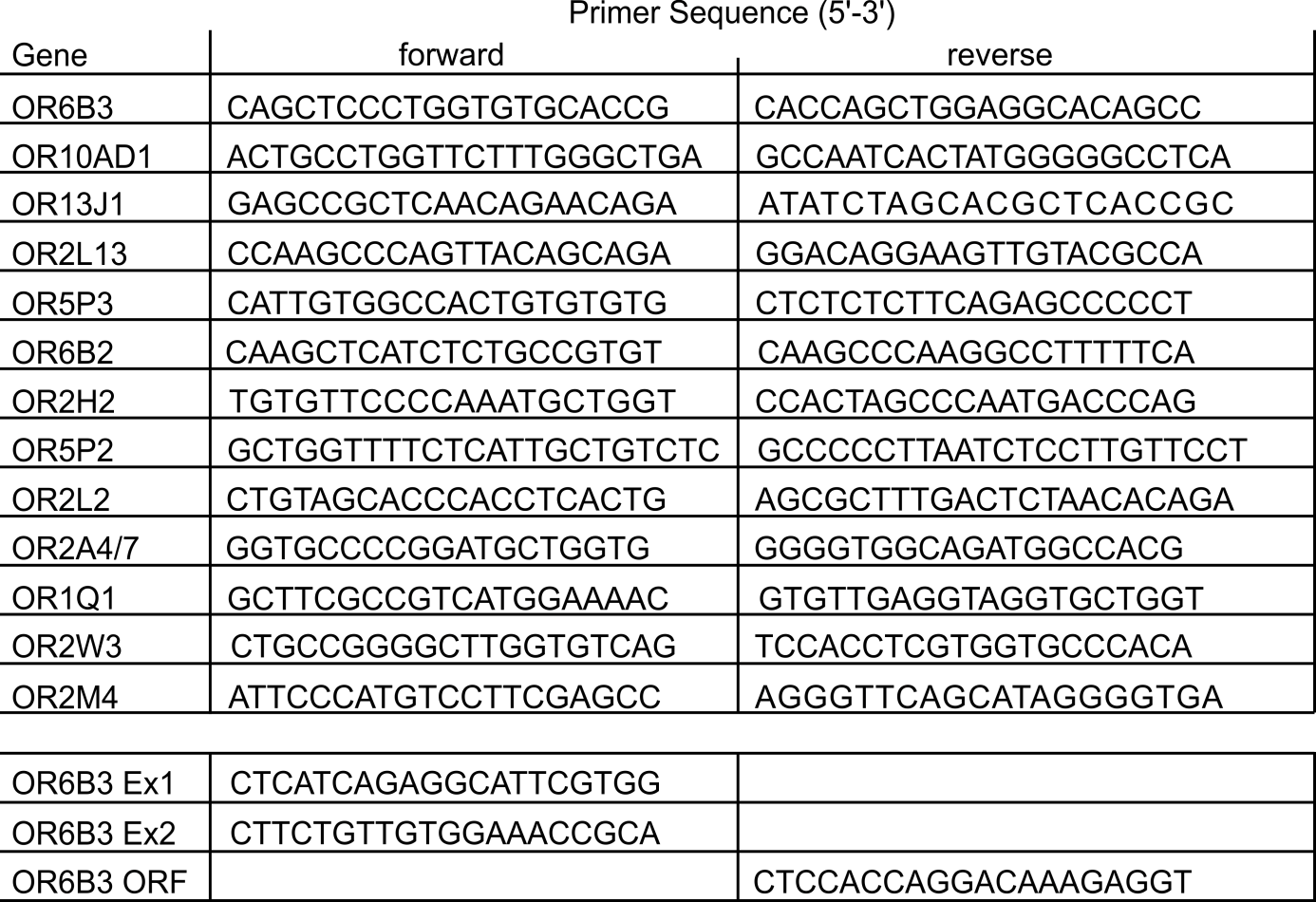


**Supplementary Table S3.** Expression of all OR genes in the human retinae. Shown are the FPKM values for all expressed OR genes in Retina1-3. For a better visualization, pseudogenes were labelled in grey. (XLSX)

**Supplemtary Table S4.** Expression of all OR genes in the humane retinae and reference tissues. Shown are the TPM values for all expressed OR genes. (XLSX)

# Supplementary Materials and Methods

**Immunocytochemical staining**

Hana3A cells were seeded on coverslips and transfected with rho-tagged OR5P3 or OR10AD1 plasmid as previously described (Zhuang and Matsunami, 2008). The cells were fixed by incubation with 4 % paraformaldehyde at 4 °C for 20 min. The specimens were washed and permeabilized in PBS+Triton X-100 (PBST). Blocking was performed in PBST+1 % gelatin and 5 % goat serum for 1 h at room temperature. The cells were then incubated overnight with the primary antibody in PBST+1 % gelatin at 4 °C. After PBST washing steps, secondary fluorescent IgGs (Life Technologies, Carlsbad, California, USA) (1:1000) and 4',6-diamidino-2-phenylindole (DAPI) were used for visualization. The secondary antibody incubation occurred for 45 min at room temperature. Afterwards, cells or tissue slices were washed with PBST and covered with Prolong® Gold Antifade reagent (Life Technologies). Micrographs were captured using a LSM510 Meta confocal microscope (Zeiss, Jena, Germany).

**Western blotting analyses**

The human donor retinae were dissected and proteins were extracted and processed for western blotting analyses as previously described (Sedmak and Wolfrum, 2010; Overlack et al., 2011). The retinal protein extracts were separated by SDS-PAGE (12% polyacrylamide gel) and transferred onto polyvinylidene difluoride membranes (Millipore, Schwalbach, Germany). Membranes were blocked using the Applichem blocking reagent (Applichem, Darmstadt, Germany) and the immunoreactivity of affinity purified rabbit polyclonal primary antibodies was detected by secondary antibodies (Alexa Flour 680; Life Technologies) using the Odyssey InfraRed imaging system (LI-COR Biosciences, Lincoln, USA).

**References**

Overlack, N., Kilic, D., Bauss, K., Märker, T., Kremer, H., van Wijk, E., et al. (2011). Direct interaction of the Usher syndrome 1G protein SANS and myomegalin in the retina. *Biochimica et biophysica acta* 1813, 1883–1892. doi: 10.1016/j.bbamcr.2011.05.015

Sedmak, T., and Wolfrum, U. (2010). Intraflagellar transport molecules in ciliary and nonciliary cells of the retina. *The Journal of cell biology* 189, 171–186. doi: 10.1083/jcb.200911095

Zhuang, H., and Matsunami, H. (2008). Evaluating cell-surface expression and measuring activation of mammalian odorant receptors in heterologous cells. *Nat Protoc* 3, 1402–1413. doi: 10.1038/nprot.2008.120
